# Intimate Partner Violence and Pregnancy: A Systematic Review of Interventions

**DOI:** 10.1371/journal.pone.0085084

**Published:** 2014-01-17

**Authors:** An-Sofie Van Parys, Annelien Verhamme, Marleen Temmerman, Hans Verstraelen

**Affiliations:** Department of Obstetrics and Gynaecology/International Centre for Reproductive Health, Faculty of Medicine and Health Sciences Ghent University, Ghent, Belgium; Vanderbilt University, United States of America

## Abstract

**Background:**

Intimate partner violence (IPV) around the time of pregnancy is a widespread global health problem with many negative consequences. Nevertheless, a lot remains unclear about which interventions are effective and might be adopted in the perinatal care context.

**Objective:**

The objective is to provide a clear overview of the existing evidence on effectiveness of interventions for IPV around the time of pregnancy.

**Methods:**

Following databases PubMed, Web of Science, CINAHL and the Cochrane Library were systematically searched and expanded by hand search. The search was limited to English peer-reviewed randomized controlled trials published from 2000 to 2013. This review includes all types of interventions aiming to reduce IPV around the time of pregnancy as a primary outcome, and as secondary outcomes to enhance physical and/or mental health, quality of life, safety behavior, help seeking behavior, and/or social support.

**Results:**

We found few randomized controlled trials evaluating interventions for IPV around the time of pregnancy. Moreover, the nine studies identified did not produce strong evidence that certain interventions are effective. Nonetheless, home visitation programs and some multifaceted counseling interventions did produce promising results. Five studies reported a statistically significant decrease in physical, sexual and/or psychological partner violence (odds ratios from 0.47 to 0.92). Limited evidence was found for improved mental health, less postnatal depression, improved quality of life, fewer subsequent miscarriages, and less low birth weight/prematurity. None of the studies reported any evidence of a negative or harmful effect of the interventions.

**Conclusions and implications:**

Strong evidence of effective interventions for IPV during the perinatal period is lacking, but some interventions show promising results. Additional large-scale, high-quality research is essential to provide further evidence about the effect of certain interventions and clarify which interventions should be adopted in the perinatal care context.

## Introduction

Intimate partner violence (IPV) is increasingly recognized as a global health problem with crucial societal and clinical implications. IPV affects women and men from all backgrounds, regardless of age, ethnicity, socio-economic status, sexual orientation or religion [1]–[3]. IPV is defined as any behavior within a current or former intimate relationship that causes physical, sexual or psychological harm, including acts of physical aggression, sexual coercion, psychological abuse and controlling behaviors [4]. It is sometimes referred to as domestic/family violence, spouse/partner abuse/assault, battering, violence against women or gender based violence. [4]–[6].

Pregnancy and childbirth are major milestones in the lives of many couples and their families. The transition to parenthood brings joy as well as new challenges to couple relationships [7],[8]. Pregnancy can be a time of particular vulnerability to IPV because of changes in physical, emotional, social and economic demands and needs. This vulnerable period, however, is not limited to the time between conception and birth. Researchers have clearly demonstrated that the risk factors for IPV associated with pregnancy encompass the timeframe of one year before conception until one year after childbirth [3],[9]–[12].

A wide range of prevalence rates, from 3 to 30% of IPV around the time of pregnancy, has been reported. Prevalence rates in African and Latin American countries are mainly situated at the high end of the continuum and the European and Asian countries at the lower end. Although estimates within regions and countries are highly variable, the majority of studies show rates within the range of 3.9% to 8.7% [2],[13]. Most studies focus mainly on physical and/or sexual partner violence, while psychological violence remains difficult to delineate and measure. Although the exact prevalence of IPV around the time of pregnancy remains unclear, it is evident that it affects a substantial group of women. In fact, IPV during the perinatal period is more common than several maternal health conditions (e.g. pre-eclampsia, placenta praevia), nevertheless IPV receives considerably less attention within perinatal care [2],[3],[14],[15].

In recent decades, research from the western world and increasingly from low and middle income countries [16] has generated growing evidence that violence is associated with detrimental effects on the physical and mental health of women, men and children [17]. IPV is associated with adverse pregnancy outcomes such as low birth weight, preterm delivery, infection, miscarriage/abortion, placental abruption, fetal injury and perinatal death. Adverse mental health consequences and behavioral risks including depression, anxiety disorders, post-traumatic stress disorder, suicide (attempts), delayed entry into prenatal care, poor maternal nutrition and use of tobacco, alcohol and illicit drugs are consistently associated with IPV around the time of pregnancy [4],[14],[17]–[29]. Most researchers and caregivers agree that perinatal care is an ideal ‘window of opportunity’ to address IPV, for it is often the only moment in the lives of many couples when there is regular contact with health care providers [2],[30]. There is a growing consensus that routine enquiry is a safe effective practice and an important first step in tackling IPV in general [24],[31]–[35]. Nevertheless, a lot remains unclear about how to deal with IPV in the perinatal care context and which interventions should be adopted.

The objective of this paper is, therefore, to provide a clear overview of the existing evidence on the effectiveness of interventions for IPV for women (and their partners/children if the intervention involves them) during the perinatal period. This review surveys randomized controlled trials (RCTs) of all types of interventions aiming to reduce IPV, and/or enhance physical and/or mental health, Quality Of Life (QOL), safety behavior, help seeking behavior, and social support.

## Methods

### Search strategy

This systematic literature review was based on an extensive search in the electronic databases PubMed, Web of Science, CINAHL, and the Cochrane Library. The search was limited to peer-reviewed articles reporting results from RCTs published in English from 2000 to 2013. The searches were systematically updated during the writing process, the last update taking place in March 2013. The following search strategy was used in PubMed: “((“violence”[MeSH Terms] OR “violence”[All Fields]) AND (“pregnancy”[MeSH Terms] OR “pregnancy”[All Fields]) AND (“Intervention (Amstelveen)”[Journal] OR “Interv Sch Clin”[Journal] OR “intervention”[All Fields])) AND (Randomized Controlled Trial[ptyp] AND (“2000/01/01”[PDAT] : “2013/12/31”[PDAT]) AND “humans”[MeSH Terms])”. The search strategy for Web of Science was: “Topic = (violence) AND Topic = (pregnancy) Refined by: Topic = (intervention) AND Document Types = (ARTICLE) Timespan = 2000–2013. Databases = SCI-EXPANDED, SSCI, A&HCI, CPCI-S, CPCI-SSH”.

We started our search in PubMed and applied the same strategy in Web of Science, CINAHL and the Cochrane Library. Reference lists of retrieved articles were checked and relevant articles were added by hand search. The database search was executed by two reviewers (ASVP & AV) independently, findings were discussed and differences resolved.

**Figure 1:**
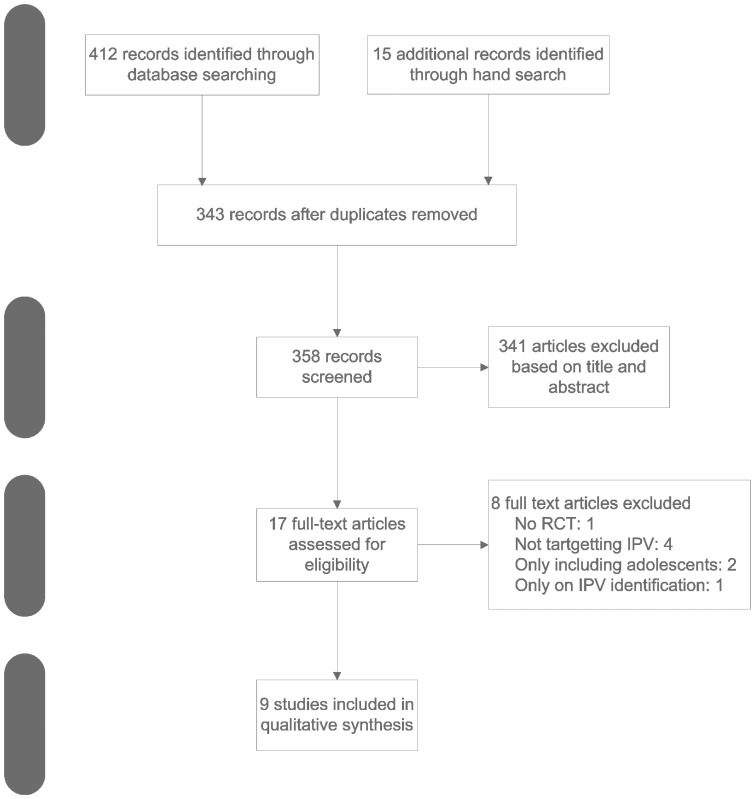
Search strategy flowchart.

Figure 1 gives a detailed overview of the search strategy.

### Inclusion criteria

Several criteria for inclusion in the systematic review were applied.

First of all, the type of participants included in the studies for this review were pregnant women of any age and/or women who had given birth in the past year (plus their partners/children if the intervention involved them).

Second, the studies had to aim at evaluating some type of intervention for IPV. Peer-reviewed papers reporting on interventions only addressing non-partner violence, reproductive coercion, child abuse/neglect, parenting, teen pregnancies, substance abuse, and disclosure of IPV were therefore excluded. Publications were also examined to ensure that they did not display the same data set as that displayed in other articles.

Third, the primary outcome of the studies had to be any measure of IPV. The secondary outcomes were physical and/or psychosocial health (e.g. pregnancy and neonatal outcome, depression, anxiety, QOL, substance use, stress), help seeking behavior, safety behavior and social support.

Fourth, we included only published RCTs, regardless of the nature, intensity or duration of the intervention, length of follow-up, or country or setting in which the participants were recruited.

### Quality assessment

After full text evaluation, the risk of bias and the quality of the selected studies was assessed by two reviewers (ASVP & AV) separately, based on “The Cochrane Collaboration's tool for assessing risk of bias” [36]. Key domains of this risk of bias assessment were sequence generation, allocation concealment, blinding, incomplete outcome data, selective outcome reporting and ‘other issues’. The reviewers independently assessed risk of bias for each study and classified every study as low, high or unclear risk of bias. Final classifications and inclusion in this review were determined by consensus. For a detailed overview of the quality assessment, see Table 1: Characteristic of the included primary studies.

**Table 1:** Characteristic of the included primary studies.

Author, Year, Country	Setting & participants (inclusion/exclusion criteria)	Intervention	Control	Outcomes & follow-up	Risk of bias
**Bair-Merritt et al., 2010 USA (Hawaii)**	643 families **Inclusion criteria:** English-speaking mother, infant at high risk for child maltreatment born between November 1994 and December 1995, not involved in child protective services.	IG = 373 families The **Healthy Start Program** (HSP). consisted of home visits by paraprofessionals providing direct services (promote child health, decrease child maltreatment by improving family functioning and reducing malleable risk factors such as IPV) and linked families to community resources.	CG = 270 families The control group participated in the HSP assessment, baseline and follow-up interviews, but did **not partake in the HSP program** (usual care).	Interviews with the infant's primary caregiver (mostly mothers): baseline interview one week after birth, follow-up interviews at the child's age of 1, 2, 3, 7, 8 & 9 years. Measures included CTS1 at baseline, CTS2 at follow-up for IPV (always past year perpetration and victimisation of physical, psychological and sexual violence). Mental Health Index (anxiety and depressive symptoms), drug and alcohol use.	Low risk
**Cripe et al., 2010 Peru**	1035 pregnant women attending antenatal care from January to July 2007 in a national referral hospital. **Inclusion criteria:** positive on AAS, Spanish-speaking, between 18–45 years old.	IG = 110 **Empowerment intervention**: abuse assessment, wallet-size referral card (listing agencies providing services to abused women) and social worker case management (this encompasses a 30 minutes supportive counselling session with education and advice in the areas of safety by a trained social worker).	CG = 110 **Standard care**: abuse assessment and wallet-size referral card.	Screening for IPV: modified AAS (physical/sexual past 12 months) Measures pre-intervention eligibility assessment and interviews between 12 & 26 gestational weeks: IPV (CTS2 past year), health-related QOL (SF-36), adaptation of safety behaviours (safety behaviours checklist), use of community resources (community resources assessment). Post-intervention interviews measures: IPV (CTS2 past year), health-related QOL (SF-36), adaptation of safety behaviours (safety behaviours checklist), use of community resources (community resources assessment).	Unclear risk
**Curry et al., 2006 USA**	1000 women attending antenatal care recruited between 2001–2003 **Inclusion criteria**: English-speaking, 13–23 weeks pregnant.	IG = 499 (only 130 women were identified as high risk due to a positive score on the AAS and/or PPP stress scale and received NCM) Referral card, offer to see video ‘Faces of abuse’, 24/7 access to the **NCM** (individualised care plan providing emotional support, basic needs assessment, assessing safety issues, discussing family concerns and providing education)	CG = 501 (101 women were identified as high risk in the CG) Offer to see video ‘Faces of abuse’. Women in the CG who screened positive for abuse, did not receive any other intervention except for a small **resource card** (except 10 women with high danger assessment scores).	All participants completed 2 research assessments, one prior to 23 weeks (T1 measures included socio-demographics, AAS and PPP) and one between 32 weeks and delivery (T2 measures included AAS and PPP.	Unclear risk
**Humphreys et al, 2011 USA**	50 women attending antenatal care from June 2006 to December 2007 **Inclusion criteria**: English-speaking, 18 years or older, <26 weeks pregnant, not first prenatal visit.	IG = 25 A 15-minutes **interactive multimedia intervention** (video doctor) and counselling. The intervention contained risk reduction messages simulating an ideal discussion with a prenatal health care provider following key principles of motivational interviewing. Messages were tailored to the participant's risk profile and intention to change. Two documents were printed automatically, cueing sheet with suggestions for counselling statements for providers and educational worksheet for participants. The intervention was designed to reduce their risks related to IPV, smoking, alcohol and illicit drugs.	CG = 25 Baseline risk assessment, no interaction with video doctor, **usual clinic care**.	Before a regularly scheduled prenatal appointment, participants completed a baseline risk assessment (socio-demographics, pregnancy history & status, tobacco, alcohol, drug use & lifetime IPV)+post-visit interview. Follow-up assessment 1 month after baseline+post-visit interview. IP(or someone important to them) V was measured through AAS (physical/sexual violence year before and since pregnancy). Other outcome measures: patient-provider discussion of IPV and perceived helpfulness.	Unclear risk
**Kiely et al., 2010 USA**	1044 women attending antenatal care from July 2001 to October 2003 **Inclusion criteria:** self-identified as minority, at least 18 years old, 28 weeks pregnant or less, Washington resident and English-speaking.	Total IG = 521 of which 169 reported IPV **Integrated cognitive behavioural intervention** delivered immediately before or after routine prenatal care (2 to 8 sessions of +/−35 minutes & up to 2 postpartum booster sessions) targeting cigarette smoking, environmental tobacco smoke exposure, depression and IPV. The intervention for IPV provided information about the types of abuse the cycle of violence, a danger assessment, and preventive options as well as the development of a safety plan and a list of community resources.	Total CG = 523 of which 167 reported IPV **Usual prenatal care**	Screening for the 4 risk factors cigarette smoking, environmental tobacco smoke exposure, depression and IPV (AAS for physical/sexual IPV previous year) Baseline interview (+/−9 days after screening): socio-demographics, reproductive history behavioural risks and CTS for frequency of physical/sexual coercion (partner to self) Follow-up telephone interviews 22–26 weeks, 34–38 weeks & 8–10 weeks postpartum: physical/sexual IPV (CTS for baseline & follow-up interviews). Data on pregnancy and neonatal outcomes were extracted from the medical records.	Low risk
**Olds et al, 2004 USA**	735 women attending antenatal care between March 1994 and June 1995 **Inclusion criteria:** no previous life births, qualified for Medicaid or no private insurance.	IG2(paraprofessional) = 245 & IG3 (nurse) = 235) The trial consists of 3 arms: control group (treatment 1), treatment 2 (paraprofessional) and treatment 3 (nurse). All arms were provided with free developmental screening and referral for children at 6, 12, 15, 21 and 24 months of age. The **home visiting program** has 3 broad goals, (1) to improve maternal and fetal health during pregnancy; (2) to improve children's health and development; and (3) to enhance mothers' personal development. The visitors helped women accomplish these goals by promoting adaptive behaviors, by helping them improve their relationships with key family members and friends (especially their mothers and boyfriends), and by promoting women's use of needed health and human services.	CG = 255 Treatment 1 (control): free developmental screening and referral for children at 6, 12, 15, 21 and 24 months of age+**usual care.**	Baseline interview (gestational age 28 & 36 weeks) and follow-up in-home assessments at 6, 12, 15, 21, 24 and 48 months of child's age 48 months (4 years) assessment (this article): Mothers reported: psychologic resources (women's intelligence, mental health and sense of mastery), number & outcomes subsequent pregnancies, socio-demographics, **physical** IPV last 2 years/past 6 months (CTS), substance use, behavior problems children. Observation mother-child interactions: home environment assessment of early learning. Children were assessed on behavioral adaptation and emotional regulation.	Unclear risk
**Taft et al., 2011 Australia**	174 women attending antenatal care from January 2006 to December 2007 **Inclusion criteria:** 16 years or older, pregnant or at least one child 5 years or younger, English- or Vietnamese speaking, disclosed IPV or were psychosocially distressed (no disclosure IPV but symptoms indicative for abuse), no serious mental illness.	IC = 113 The women in the IC received a resource card and up to 12 months support from **non-professional mentor mothers** providing: non-judgmental support, assistance in developing safety strategies, a trusting relationship, information and assistance in referral to community services.	CG = 61 Women in the CG received a resource card and **usual care.**	Baseline & 12 month follow-up questionnaires used the following measures: CAS for IPV (emotional/physical/sexual), EPDS for depression, SF-36 for general health & well-being, PSI-SF for parenting stress, MOS-SF for social support.	Unclear risk
**Tiwari et al., 2005 Hong Kong**	110 women attending antenatal between May 2002 and July 2003. **Inclusion criteria:** over 18 and less than 30 weeks pregnant and attending first antenatal appointment.	IC = 55 **Empowerment intervention**, a 30 minutes one-to-one session (at enrolment) including advice in the areas of safety, choice making, problem solving and empathic understanding. A brochure reinforcing the information was given after the session.	CG = 55 **Standard care:** a wallet size resource card after enrolment.	Screening: AAS (physical/sexual/emotional-psychological male partner abuse last year) Enrolment: CTS, SF-36 & demographics Telephone follow-up interview 6 weeks postpartum: CTS, SF-36 & EPDS.	Low risk
**Zlotnick et al., 2010 USA**	54 women attending antenatal care **Inclusion criteria:** attending prenatal care visit, between 18 and 40 years of age, no DSM-IV Axis 1 disorder.	IC = 28 The intervention consisted of four **60 minutes individual sessions** over a 4 week period before delivery and 1 booster session within 2 weeks of delivery. The content of these sessions was based on the principles of interpersonal psychotherapy emphasizing social support. Following topics were a.o. covered healthy/abusive relationships, disputes, resolving interpersonal conflicts stress management skills, safety plan, “baby blues,” and postpartum depression, PTSD, substance use, transition to motherhood, self-care and social support networks.	CG = 26 **Standard (medical)** care, the educational material and a listing of resources for IPV.	Screening: CTS2 (past year physical/psychological/sexual IPV)and demographics Baseline assessment: current affective disorders, PTSD and substance use (SCID-NP). Assessments administered at intake, 5–6 weeks after intake, 2 weeks after delivery, 3 months postpartum): CTS2 (past year or since the last assessment physical/psychological/sexual), LIFE (assess major depressive disorders and PTSD), EPDS (depression level), Davidson trauma scale (PTSD), criterion A from the PTSD module of the SCID-NP (history of trauma).	Unclear risk

Legend:

AAS = Abuse Assessment Screen.

CAGE = Cut down, Annoyed, Guilty, Eye-opener (alcoholism screening tool).

CAS = Composite Abuse Scale.

CG = Control Group.

CTS = Conflict Tactics Scale.

CTS2 = revised Conflict Tactics Scale.

EPDS = Edinburgh Postnatal Depression Scale.

IG = Intervention Group.

IRR = Incidence Rate Ratio.

LIFE = Longitudinal Interval Follow-up Examination.

MOS-SF = Medical Outcomes Scale - Short Form.

NCM = Nurse Case Management.

MCS = Mental Components Scores (SF36).

PCS = Physical Components Scores (SF36).

PPP = Prenatal Psychosocial Profile.

PSI-SF = Parenting Stress Index – Short Form.

SCID-NP = Structured Clinical Interview for the DSM-IV Axis Disorders – Nonpatient Version.

SF36 = Short Form Health Survey.

SVAWS = Severity of Violence Against Women Scale.

### Data extraction

Using a specially designed data extraction form, the two reviewers independently extracted information from the selected papers. Data items compromised country, setting, sample size & participants, sampling methods, measuring tools, description of the intervention and control group(s), outcomes, and follow-up period. Authors were contacted if additional information was required.

Initially, we planned a meta-analysis to quantify and compare the interventions identified. Unfortunately it was not feasible to perform a meta-analysis due to the limited amount of data and the large variation in interventions, outcome measures and measurement time points.

The PRISMA guidelines were used as a framework for this review [37].

## Results

Through our electronic database search, we retrieved 412 potentially relevant articles based on keywords and limits set (60 in PubMed, 343 in Web of Science, seven in CINAHL and two in the Cochrane database). Fifteen additional articles were identified through hand search. After title and abstract evaluation, 69 duplicates were removed, leaving 343 to be included in the next step. Thereafter, out of 358 articles (343+15 articles retrieved through hand search) screening resulted in 17 articles deemed eligible for more detailed evaluation. After full text evaluation another eight were excluded because they did not meet the inclusion criteria, leaving nine studies submitted to critical appraisal and included in this systematic review [17],[38]–[45]. Details on setting/participants, intervention/control activities and outcomes are given in Table 1: Characteristic of the included primary studies.

Out of these nine studies, six were conducted in the USA, one in Peru, one in Australia, and one in China. All studies recruited participants through hospital-based antenatal care, with sample sizes ranging from 50 to 1054 women.

Three studies measured the impact of a home visitation program involving paraprofessionals (non-professionals trained to do the home visits and deliver the intervention), mentor mothers (lay mothers trained to do the home visits, provide peer support and mentoring), and/or nurses and followed participants for between one up to nine years.

The six other studies evaluated the effect of some form of supportive counseling, varying from one 30-minute session up to six 60-minutes sessions or 24/7 access to a Nurse Case Manager (NCM). Most (n = 6) of the interventions were specifically designed to target IPV as the main objective, but some (n = 3) were part of a larger, multifaceted intervention in which IPV was one of the targets parallel to e.g. smoking, depression, child health, parenting. All studies compared the intervention to usual or standard care, which, due to ethical considerations, generally implied that patients were provided a referral card or a list of community resources.

Throughout the rest of this paper the term IPV will be used to refer to the combination of physical and sexual and psychological partner violence, unless specified otherwise.

### Home visitation programs

#### Primary outcome

After three years of program implementation Bair-Merritt et al. [41] found that, intervention women reported a lower, albeit statistically marginally non-significant, adjusted rate of IPV victimization [Incidence Rate Ratio (IRR) 0.86, 95% CI, 0.73–1.01] and a significantly lower rate of perpetration (IRR 0.83, 95% CI, 0.72–0.96) than the control group. Intervention women showed significantly lower rates of physical assault victimization (IRR 0.85; 95% CI, 0.71–1.00) and significantly lower perpetration (IRR 0.82, 95% CI, 0.70–0.96). Although rates of overall IPV victimization and perpetration were also lower after 9 years, these results were not statistically significant. In other words, perpetration rates decreased significantly and victimization rates showed a trend towards decrease after three years, but not after nine years.

Olds et al. [44] found on the one hand, no adjusted statistically significant effects of **paraprofessional visits** on the experience of physical partner violence in the intervention group (IG) versus the control group (CG) (14.2% vs. 13.6%, *P* = 0.88, OR 1.05, 95% CI not reported) in the six months prior to four year follow-up. On the other hand, **nurse-visited** women did report (6.9% vs. 13.6%, *P* = 0.05, OR 0.47, 95% CI not reported) a significant decrease in physical partner violence.

Taft et al. [39] reported evidence of a true difference in mean abuse scores at 12 months follow-up (15.9 vs. 21.8, AdjDiff −8.67, 95% CI, −16.2–−1.15, *P* = 0.03).

#### Secondary outcomes

In the study of Olds et al. [44], women visited by **paraprofessionals** reported a statistically significant greater sense of mastery (101.25 vs. 99.31, *P* = 0.03) and better mental health (101.21 vs. 99.16, *P* = 0.03) than control subjects, had fewer subsequent miscarriages (6.6% vs. 12.3%, *P* = 0.04, OR 0.5, 95% CI not reported), and fewer low birth weight newborns (2.8% vs. 7.7%, *P* = 0.03, OR 0.34, 95% CI not reported). There were no statistically significant effects of **nurse visits** on these variables

Taft et al. [39] reported a trend favoring the intervention regarding depression (19/85 vs 14/43; AdjOR 0.42, 95% CI 0.17–1.06), physical wellbeing mean scores (AdjDiff 2.79, 95% CI, 0.40–5.99), and mental wellbeing mean scores (AdjDiff 2.26; 95% CI, 1.48–6) but no observed effect on parenting stress.

### Supportive counseling

#### Primary outcome

The women in the intervention group of Kiely et al. [40] experienced statistically significant fewer recurrent episodes of IPV during pregnancy and postpartum than women receiving usual care (adjOR 0.48, 95% CI, 0.29–0.80). Those with minor IPV were significantly less likely to experience further episodes during pregnancy (first follow-up 22–26 gestational weeks OR 0.48, 95% CI, 0.26–0.86; second follow-up 34–38 gestational weeks OR 0.53, 95% CI, 0.28–0.99) and postpartum (OR 0.56, 95% CI, 0.34–0.93). Those with severe IPV showed significantly reduced episodes only during postpartum (OR 0.39, 95% CI, 0.18–0.82). Women experiencing physical IPV showed a significant reduction in such violence at the first follow-up (OR 0.49, 95% CI, 0.27–0.91) and postpartum (OR 0.47, 95% CI, 0.27–0.82). For sexual IPV the intervention did not significantly reduce episodes of violence at any point in time.

Tiwari et al. [45] reported statistically significant less psychological [Mean Difference (MD) −1.1, 95% CI, −2.2 to −0.04)] (but not sexual) abuse and significantly less minor (MD −1.0, 95% CI, −1.8 to −0.17) (but not severe) physical violence in the intervention group.

Cripe et al. [17] reported no statistically significant differences in the occurrence of IPV between the intervention and control groups after an empowerment counseling session.

Curry et al. [42] did not report any results on IPV, nor were the authors able to provide the IPV data we requested.

Humphreys et al. [43] found no statistically significant differences in prevalence of physical and/or sexual partner violence between the two groups at baseline and did not report partner violence after intervention.

The intervention by Zlotnick et al. [38] did not significantly reduce the likelihood of IPV during pregnancy or up to three months postpartum.

#### Secondary outcomes

Women in the IG of Kiely et al. [40] had significantly fewer very preterm neonates (1.5% vs. 6.6%, *P* = 0.03) and an increased mean gestational age (38.2±3.3 vs. 36.9±5.9, *P* = 0.016).

Tiwari et al. [45] reported significantly higher physical functioning in health related QOL (MD 10, 95% CI, 2.5–1.8) and a significant reduction of role limitation due to physical problems (MD 19, 95% CI, 1.5–37) and emotional problems (MD 28, 95% CI, 9.0–5.0). There was, however, also more bodily pain in this group (MD −1.3, 95% CI, −23–−2.2). Significantly fewer women in the IG reported postnatal depression at follow-up (RR 0.36, 95% CI, 0.15–0.88).

Curry et al. [42] found no statistically significant decrease of total stress scores between the two groups, although total stress scores of both intervention and control women significantly decreased (*P*<0.001) between follow-up periods.

The intervention by Zlotnick et al. [38] did not significantly reduce the likelihood of a major depressive episode or post traumatic stress disorder (PTSD). They found a trend towards decrease during pregnancy but not during postpartum.

Cripe et al. [17] found a trend towards improved QOL, safety and help seeking behaviors (church and police) in the IG, but no statistically significant differences between the two groups.

**Figure 2:**
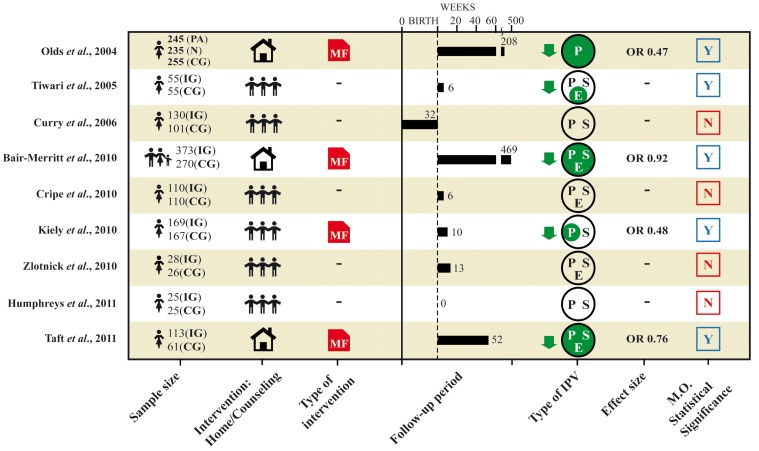
Overview results. PA = Paraprofessional. N = Nurse. IG = Intervention Group. CG = Control Group. MF = Multifaceted intervention. P = Physical. S = Sexual. E = Emotional. M.O. statistical significance = statistical significant results of measured primary outcome.

The following figure 2 gives illustrates the correlations between the type of intervention and the impact on the reduction of IPV.

## Discussion

The results of our systematic review demonstrate that there are few RCTs evaluating interventions for IPV during the perinatal period. Moreover, the overall quality of the nine studies identified is limited and did not produce strong evidence that certain interventions are effective. This finding is also endorsed by Jahanfar et al. [46]. The evidence of IPV interventions outside the context of pregnancy remains similarly insufficient and inconclusive 24,[31],[47]–[49].

Nevertheless, five out of nine studies in our review reported a statistically significant decrease in some form of IPV (odds ratios from 0.47 to 0.92). The most promising results identified by this review are to be found in the home visitation programs and multifaceted counseling-interventions. The three studies [39],[41],[44] on **home visitation programs** all showed a statistically significant decrease in IPV victimization (and one in perpetration). However, although Olds et al. [44] noted a significant decrease in physical IPV for the nurse-visited women, this was not found for the paraprofessional-visited women. The authors attributed this finding to an increased emphasis among the nurses on partner violence, but it remains unclear if this was really the case. With regard to the secondary outcomes, Olds [44] reported significantly better mental health, fewer subsequent miscarriages and low birth weight newborns in the paraprofessional-visited but not in the nurse-visited women. The different impact of nurses and paraprofessionals raises questions about the mechanisms through which the interventions affected the outcomes.

It is interesting to note that out of six studies evaluating different types of **supportive counseling**, only two reported a statistically significant effect of the intervention on IPV. First, the high-quality study by Kiely et al. [40] found that their cognitive behavioral intervention significantly reduced recurrent episodes of IPV (except for sexual IPV). Second, Tiwari et al. [45] reported significantly less psychological and minor physical (except for sexual IPV) violence in the intervention group. Sexual partner violence seems to be a form of violence that is difficult to influence. The other four studies [17],[38],[42],[43] did not find a significant difference in IPV between the intervention and control groups. Concerning secondary outcomes, Kiely et al. [40] observed significantly fewer very preterm neonates and an increased mean gestational age in the intervention group. Tiwari et al. [45] reported significantly fewer women with postnatal depression and improved QOL in the intervention group.

None of the studies reported any evidence of a negative or harmful effect of interventions, although only one study [43] mentioned assessing potential harms caused by intervention.

The results should be interpreted with caution and within the light of serious methodological challenges. Researching violence is inherently associated with numerous ethical and safety issues, making it very difficult to produce strong evidence. We identified considerable variation in categorizing certain behavior as IPV, research settings, study populations, sample sizes, content of the intervention, and length of follow-up. Intrinsic to the difficulties associated with the study subject sample sizes are small, there is a considerable loss to follow-up, and it is impossible to blind respondents. Moreover, few studies adjusted their analysis for confounding factors (e.g. childhood abuse), which can create an oversimplified image of reality. However, it should be remembered that lack of statistically significant results does not necessarily imply clinical irrelevance. Some interventions might be effective but not have reached significance level due to methodological and/or ethical challenges.

It is striking that five out of the nine studies reported decreases in IPV after a certain point in time but that these decreases did not significantly differ between intervention and control groups. Apparently, with time (certain) wounds heal. However, other explanations can also be hypothesized.

First, as far as we know, in all the studies reviewed, identifying IPV was not part of routine perinatal care but an additional research-related activity (also known as the Hawthorne-effect) [31],[34]. Asking IPV-related questions to women in the control group, mostly in combination with handing out a referral card could have had a larger impact than assumed. McFarlane et al. [30] found that “simple assessment of abuse and offering referrals has the potential to interrupt and prevent recurrence of IPV”. In other words it is possible that the ‘intervention’ in the control group is more effective than anticipated and therefore no clear difference between the two groups is detected.

Second, it seems reasonable to question the legitimacy of using IPV as a main outcome measure. Given the complexity of intervening factors between identification and IPV reduction (with many not under the control of health care providers), interventions should not necessarily be expected to decrease IPV [35]. Internal changes (mental health, QOL, …) are potentially more informative for evaluating the impact of an intervention for IPV. Significant changes in active or passive experiences of violence may not be observable for some time [5],[47],[48],[50]. At the time of measurement, respondents might simply not acknowledge the violence, or be ready to make changes or accept help. Some counseling interventions (developing safety plans, seeking help, …) might come too early and/or are not adapted to specific needs and therefore prove ineffective [51],[52]. In this review, we identified only one study [43] that included some measure of ‘readiness to change’ which might have contributed to the lack of significant results.

Furthermore, our systematic review yielded only one study [41] reporting both maternal victimization and perpetration behavior, in which there is the striking observation that the rate of perpetration acts in women was twice as high as the victimization acts in both intervention and control groups (at baseline). The intervention seemed to reduce mainly maternal perpetration behavior, but paternal victimization nor perpetration behavior was not directly measured. This finding adds to the debate on gender symmetry in the perpetration of violence and the discussion about over-disclosure by women and under-disclosure by men. Yet, pregnant women's use of violence is virtually ignored by most authors [3]. Moreover, Hellmuth et al. [53] found that IPV perpetration during pregnancy and/or postpartum is associated with negative health outcomes. Therefore, measuring only subjection to violence as a measure of effectiveness of an intervention seems quite insufficient. More attention should be given to outcome measures reflecting the complex process of changing destructive interaction dynamics.

We are aware that this systematic review has several limitations. The choice of databases, inclusion criteria, risk of bias assessment, and interpretation of results all required the individual judgment of the authors. We took various steps to minimize bias at all stages of the review process, but a different review team may not fully agree with our assessment.

## Conclusion

This systematic review indicates that strong evidence of effective interventions for IPV during the perinatal period is lacking. Nonetheless, home visitation programs and some multifaceted counseling interventions produced promising results. It is obvious that additional large-scale, high-quality research (with meta-analysis) is essential to tackle the remaining questions and provide further evidence about the effect of certain interventions. Future research should focus on several levels simultaneously (individual, relations, community, and society). Intervening in a single risk factor may be unsuccessful because other risk factors may persist as barriers to the desired change. Readiness to change, help seeking strategies and the complex mutuality of IPV should be taken into account. Serious thought should be given to appropriate outcome measures and to including process indicators in evaluating effectiveness.

## Supporting Information

Checklist S1 **PRISMA checklist.**
(PDF)
